# Fast Degradation of Solid Electrolyte in Initial Cycling Processes, Tracked in 3D by Synchrotron X‑ray Computed Tomography

**DOI:** 10.1021/acsnano.4c17739

**Published:** 2025-05-28

**Authors:** Shuai Hao, Sohrab R. Daemi, Thomas M. M. Heenan, Wenjia Du, Malte Storm, Mohamed Al-Hada, Christoph Rau, Dan J. L. Brett, Paul R. Shearing

**Affiliations:** ‡ Electrochemical Innovation Lab, Department of Chemical Engineering, 4919 University College London, London WC1E 7JE, United Kingdom; § The Faraday Institution, Quad One, Harwell Science and Innovation Campus, Didcot OX11 0RA, United Kingdom; ∥ 120796 Diamond Light Source Ltd, Harwell Science & Innovation Campus, Didcot OX11 0DE, Oxfordshire, United Kingdom; # The ZERO Institute, 98956 University of Oxford, Holywell House, Osney Mead, Oxford OX2 0ES, United Kingdom

**Keywords:** Solid electrolyte, degradation, cracks, in situ X-ray CT, morphology

## Abstract

Solid-state lithium batteries are developing rapidly as a promising next-generation battery, while challenges still persist in understanding their degradation processes during cycling due to the difficulties in characterization. In this study, the 3D morphological evolution of the Li_3_PS_4_ solid electrolyte was tracked during electrochemical cycles (plating and stripping) until short circuit by utilizing in situ synchrotron X-ray computed tomography with sufficient spatial and temporal resolution. During the degradation process, cracks in the electrolyte alternately generated from the two electrode/electrolyte interfaces and propagated until shorting. The lithium dendrites filled in the electrolyte cracks but had a greatly reduced filling ratio after the first plating stage; therefore, the cell could continue working for some time after the solid electrolyte was fully fractured by cracks. The compression of the two lithium electrodes mainly occurred in initial cycles where a ca. 4–7 μm reduction in thickness was observed. The mechanical force and electric potential fields were modeled to visualize their redistributions in different stages of cycling. The release of strain energy after the first penetration and thereafter the subsequent driving forces are discussed. These results reveal a fast degradation of solid electrolyte in the initial cycles, providing insights for further modifications and improvements in solid-state batteries.

## Graphical abstract

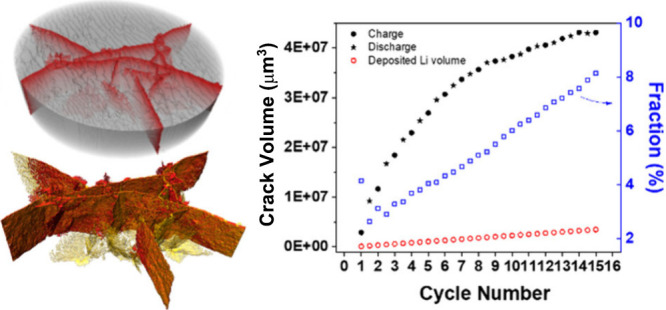

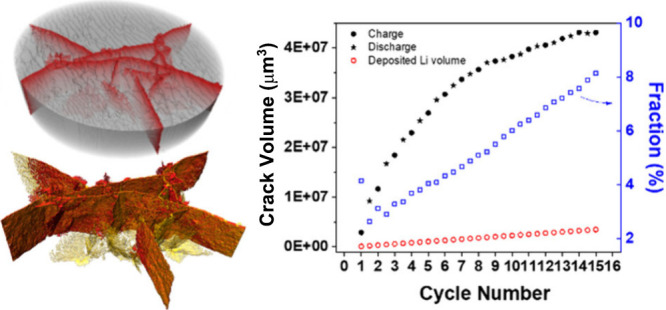

## Introduction

With increasing demands on commercial lithium-ion batteries in electric vehicles, solid-state lithium batteries have attracted great interest from both industrial and academic researchers. They have unique advantages in safety and energy densities because of the nonflammable solid electrolytes and the high-capacity lithium metal anode. However, several obstacles seriously hinder the implementation of solid-state batteries, including the relatively low ionic conductivity of the solid electrolyte,^1^ the interface between electrodes and solid electrolytes,^2^ as well as the risk of internal short circuit caused by dendrite growth^3^ (the lithium distributions in solid electrolyte are not classically “dendritic” from a geometrical definition, so we use lithium deposition/penetration in the following). In addition, compared with the liquid electrolyte system, the mechanical forces arise as a more critical problem due to the limited available space to accommodate the volume changes during charging/discharging.

In a degradation analysis of the solid-state battery, the solid electrolyte is the location where the lithium penetration, mechanical cracking, and interfacial degradation occurs. Therefore, understanding its morphological evolution during electrochemical cycling is critical to understanding degradation processes. However, it is challenging to directly observe solid electrolytes, especially their internal or interfacial changes, due to their opacity and the buried nature of interfaces in intimate contact with other solid electrodes. Deposited lithium and cracks within SSEs are completely buried and are therefore inaccessible to optical microscopy (OM) or scanning electron microscopy (SEM). In many cases, the solid electrolytes have to be disassembled from the batteries and fractured to expose the internal cross sections before testing under OM^4^ or SEM.^5–7^ However, the structures of fragile deposited lithium and cracks could be disturbed during these preparation processes and the solid electrolytes (particularly sulfides) can rapidly undergo side-reactions with air.^8,9^ Utilizing characterization methods with penetrative ability, such as nuclear magnetic resonance^10^ and neutron depth profiling,^11,12^ only 1D spectra can be obtained within the constraints of conventional cell designs. Focused ion beam (FIB) microscopy can provide a 3D image^13–15^ but may also disturb the local mechanical environment through ion beam milling; besides, the sample volume may not be large enough to provide a statistically representative view of the bulk electrolyte as it is typically limited to tens of cubic micrometers. Furthermore, to characterize the dynamic changes of the solid electrolyte during a degradation process, real-time observation is necessary but more difficult. In specially designed batteries, in situ OM^4,14,16–18^ and in situ SEM^19–21^ tests were conducted but are limited to the sample surface or a cross section.

X-ray computed tomography (CT) provides a technique to detect the internal structures of SSBs nondestructively and in 3D. In previous works, the pore structures and distributions in Li_7_La_3_Zr_2_O_12_,^22^ Li_10_GeP_2_S_12_,^23^ and Li_5.3_PS_4.3_ClBr_0.7_ ^24^ were clearly imaged before and after cycling. Lithium protrusions through polymer electrolytes^25^ and the gradual loss of contact at interfaces between lithium and Li_6_PS_5_Cl^26^ have also been observed with the help of this method. The high flux and brilliance X-rays available at synchrotron facilities (such as the Diamond beamline I13-2 ^27^) can substantially reduce exposure times, which makes dynamic studies possible.^28–30^ The whole solid electrolyte can be captured in a lager field of view compared with FIB studies. In our previous work, we optimized the material selection, battery case, and scanning parameters, so that a voxel resolution of 0.8 μm was realized in in situ synchrotron X-ray CT. Then, the morphological evolution of deposited lithium and cracks inside Li_3_PS_4_ was successfully tracked during repetitive plating^29^ (called “repetitive charging” in the following, in contrast to “cycling”.). In our previous studies, the scenario of *cycling* (ie. plating *and* stripping) until short circuit was not explored, but this is clearly more representative in practical applications.

In this work, the morphological changes inside an intact solid electrolyte were captured during extended cycling until short circuit, so that the whole degradation process was tracked. Three-dimensional images were reconstructed in each step. The cross-sectional images of Li/Li_3_PS_4_ interfaces were extracted to observe their degradation. We calculated the speed of crack propagation, the changes of electrode thickness, and the filling ratio of lithium deposition in cracks. We found that the major changes were largely confined to the initial stage. Furthermore, different distributions of the mechanical force and electric potential field were modeled based on time-series images. The first release of strain energy and subsequent driving processes are discussed in detail.

## Results and Discussion

In order to maximize image quality and resolution, Li_3_PS_4_ (LPS) was selected as a model solid electrolyte because of its lower X-ray absorption coefficient compared with oxide materials (e.g., Li_7_La_3_Zr_2_O_12_) and other sulfide (e.g., Li_10_GeP_2_S_12_) electrolytes. By pressing into a small pellet with a diameter ca. 2 mm, the LPS was sandwiched with two lithium electrodes, then assembled into a custom Swagelok battery. The impact from the cathode was excluded to focus on the interactions of solid electrolyte and Li anode. The battery was fixed onto the sample holder on the synchrotron beamline and connected to a potentiostat (Gamry, Interface 1000E) onsite, so that any movement or disturbance was minimized during tracking the morphological evolution. During the in situ test, the cell was repeatedly charged and discharged for 30 min, while at the end of each step the bias voltage was removed and X-ray radiographic projections were captured while rotating the cell through 180°. After counter rotation, back to the original position, the cell continued the next charging/discharging step. The experimental setup is schematically shown in Figure 1a, and more details are contained in the Experimental Section.

**Figure 1:**
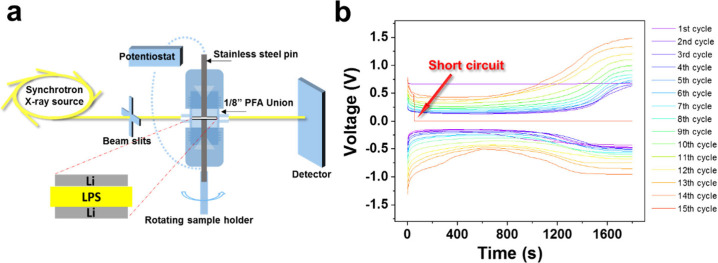
(a) Schematic of Swagelok cell; (b) galvanostatic cycling for 15 cycles until short-circuit.

With the help of the high-brilliance synchrotron X-ray source as well as the battery configuration and material selection, a higher resolution of 0.8 μm (voxel size) was obtained compared with previous reports.^23,28,31–33^

Figure 1b shows the galvanostatic cycling curves of the Li/LPS/Li cell. Overall, the polarization-voltage gradually increased during the cycling processes, which also increased slightly during individual charging/discharging steps. Similar phenomena have also been observed in Li_10_SnP_2_S_12_ ^28^ and Li_1+*x*_Al_*x*_Ge_2–*x*_(PO_4_)_3_.^31^ This indicates a growing impedance in the whole cell which is caused by several processes, including decreased contact areas at the Li/LPS interface, a large number of cracks formed inside the solid electrolyte and a gradual release of stack pressure. These will be discussed further in the following sections. In the first charging cycle, the overpotential was slightly higher due to the imperfect connections in the cell configuration. In the 15th charging cycle, the voltage abruptly dropped to 0 V, which indicated the occurrence of short circuit. While operational batteries have been inspected in situ in previous works,^31,32^ here, a complete cycling process until short circuit was tracked, which is considered more representative of conditions in practical applications.

By comparing the same slices in LPS after different charging/discharging times, the morphological evolution in solid electrolytes during the whole cycling process is clearly shown in Figure 2 and Videos 1 and 2. Figure 2a illustrates the spatial locations of these orthogonal slices (named as “top view” and “side view”) inside the pellet. Corresponding to the current direction, the lithium electrode on top is referred to as the “anode”, while the one at the bottom is “cathode” (Figure 2a, f–i, n–q). Note the top-view slice was very near to the LPS/cathode interface (Figure 2b–e, j–m). Additionally, another slice was extracted in the middle of the pellet (Figure S1). Based on the Beer–Lambert law, different X-ray linear attenuation coefficients of materials result in a different gray scale in the reconstructed slices. In this case, high grayscale signifies LPS with high attenuation and low grayscale signifies lithium metal and cracks (empty space) with low attenuation.

**Figure 2:**
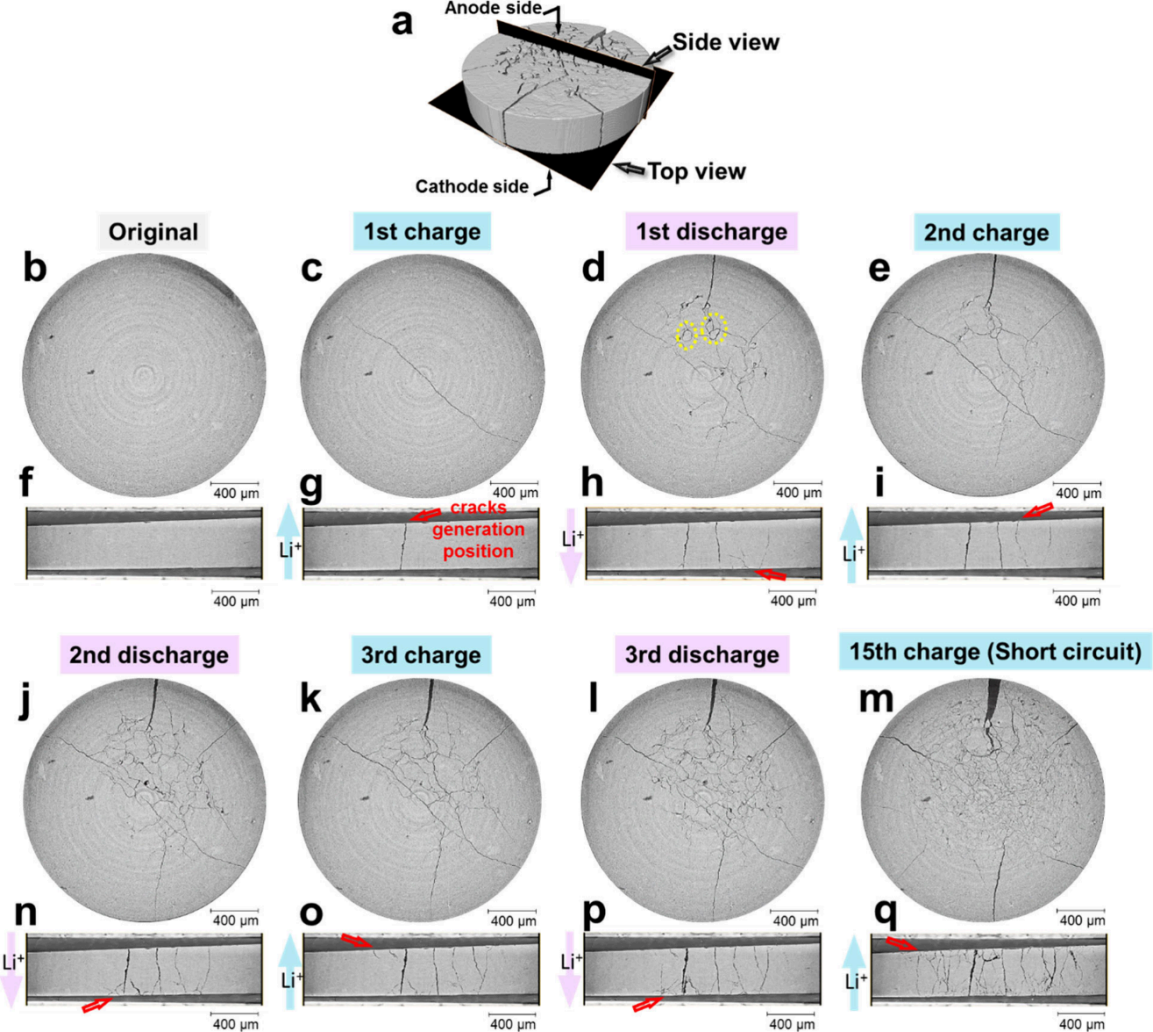
2D orthogonal slices (top view: b–e and j–m; side view: f–i and n–q) extracted from 3D tomogram (as position illustrated in panel a, the 3D rendered volume of LPS pellet after 15th charging), comparing them at different states of cycling: original state (b, f), after the 1st charging (c, g) and discharging (d, h), after the 2nd charging (e, i) and discharging (j, n), after the 3rd charging (k, o) and discharging (l, p), and after the 15th charging (m, q). The positions of crack generation are indicated by red arrows.

As shown in Figure 2b,f, the original LPS pellet was intact without detectable cracks before cycling. All subsequent morphological changes were therefore induced by the electrochemical process, rather than a pure mechanical force from the battery case itself. After the first charging (Figure 2c,g), one crack formed immediately and traversed the pellet. Based on our recent work,^29^ the crack originated from the anode side after lithium was deposited there. When the current was reversed in the first discharging (Figure 2d,h), several new cracks formed from the cathode side, as indicated by the red arrows. In the top view in Figure 2d, new cracks horizontally spread into branches toward different directions and formed some small “rings” at connection points, as indicated by the dashed yellow annotation. After the first charging, these “rings” were also observed to preferentially locate near the anode side, as shown in Figure S2, indicating that cracks tended to initiate in a ring-like geometry near the interface where the lithium is deposited. The 3D morphology of these “rings” will be discussed in the following sections.

In the second and third charging/discharging and the subsequent cycling steps before short circuit, cracks alternately generated from the anode and cathode side of the LPS pellet, as notably displayed in Video 2 and indicated in the side views (Figure 2i,n–p). Most of the cracks grew perpendicularly to the pellet surface and connected to each other in the middle of the pellet. Observing the top views near the cathode side in Video 1 and Figure 2b–e,j–m, cracks were found to slightly shrink every time after charging; they then expanded (or “recovered”) following the next discharge. By contrast, this kind of repeated shrinkage and expansion is less obvious at the middle of the pellet (Figure S1). Consequently, the LPS pellet was “bent” back and forth as a whole. This phenomenon was related to the relatively rigid body of LPS (Young’s modulus of 25 GPa,^34^ hardness of 1.9 ± 0.2 GPa, and fracture toughness of 0.23 ± 0.04 MPa m1/2 ^35^), which would be significantly different from the soft polymer electrolytes and composite electrolytes. The mechanical properties of solid electrolytes largely influence their behavior under the coupled electro-chemo-mechanical force fields.

Finally, when the cell was short-circuited (after the 15th charging), a large number of cracks formed and spread throughout the pellet (Figure 2m,q, Figure S1i). Comparing Figure 2m with Figure S1i, the cracks near the cathode side were narrow and formed numerous small “rings”; they became wider and connected into several straight branches in the middle of the pellet. This is also reflected in the side view (Figure 2q) as the cracks have many narrow branches near the top and bottom surfaces of the pellet, but wide and straight branches in the middle. Video 2 clearly reflects the process: in each step, new cracks originated as small branches from the interfaces, then merged into wider ones in the middle of pellet. Besides, because cracks interrupted the conduction paths for lithium ions in the solid electrolyte, the impedance of the cell kept increasing as the number of cracks increased.

Figure 3 shows the magnified LPS/anode interfaces in side-view: before cycling, the surface of the LPS pellet was flat and smooth and closely contacted with the lithium electrode under stack pressure. As indicated by the arrow, only one small void was found. After the first charging and discharging, another two voids formed, then gradually merged with the first one into a long void after the fourth discharge. Along with the cracks generated, the voids always expanded after charging and shrank slightly after discharging. Finally, there were several voids at the interface after short circuit, as well as some small LPS “shards” caused by cracks. As a result, the pellet surface was not as flat as the original state but roughened visibly. Similarly, the interface between the LPS and cathode also formed voids and shards gradually, losing local contact at these interfaces. The decreasing contact area caused higher current density which was a trigger for generating lithium dendrites/deposition. This phenomenon has been also observed in Li_6_PS_5_Cl,^26^ Li_6.25_Al_0.25_La_3_Zr_2_O_12_,^36^ and in sodium batteries.^37^

**Figure 3:**
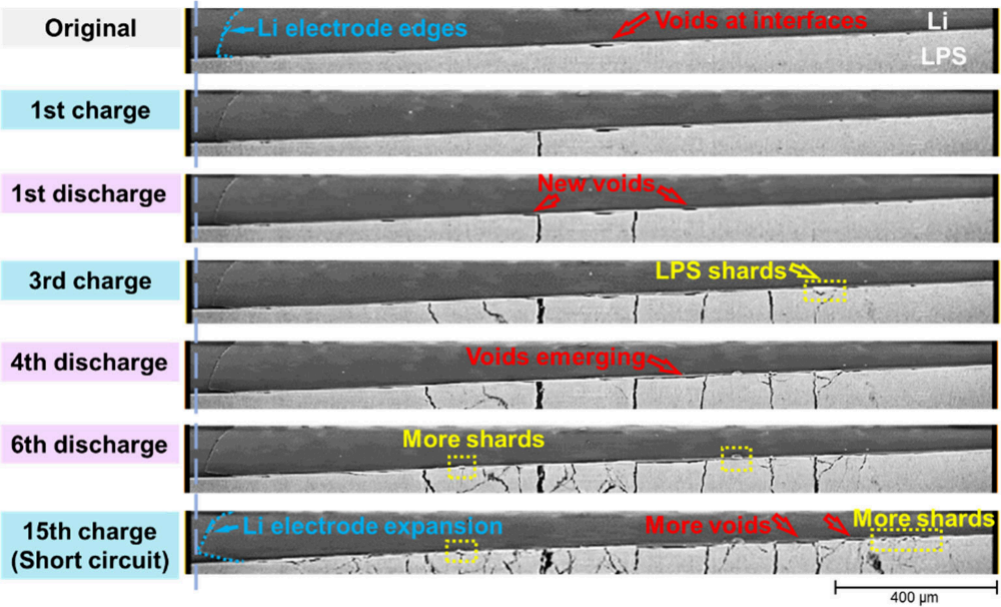
Magnifications of the side view at the interfaces between the LPS pellet and anode lithium electrode after different cycling steps.

It is worth noting that, although cracks already penetrated through the solid electrolyte after the first charging, the cell was not short-circuited immediately but worked well with stable voltage curves for 14 subsequent cycles. This will be analyzed further in the following section.

To better reveal the morphological evolution inside the solid electrolyte during cycling, Figure 4 shows the 3D structure of the cracks after different cycling steps. In order to clearly observe the propagation steps, the cracks newly generated after each progressive cycle are colored in transparent yellow, and the previously formed parts are in red. As shown in Figure 4a, the crack generated during the first charging is in a thin sheet and spread perpendicularly to the pellet surface. Near the interface with the anode Li, there is an approximate semiellipsoidal feature, as indicated by an arrow. It was formed by the “rings” visualized in the 2D slices (Figure S2). After the first discharging (Figure 4b), the first crack continued growing laterally to reach the pellet edge. Simultaneously, another three or four branches generated from the cathode side and propagated in different directions. In this step, the semiellipsoidal features appeared near the cathode side. During the second charging/discharging (Figure 4c,d) and the rest of cycling, as the current direction was repeatedly reversed, new cracks generated alternately from the interface with the anode or cathode. Correspondingly, new semiellipsoidal features, as initiation points of cracks, formed near the interfaces, which are indicated by arrows in Figure 4a–d. Meanwhile, as the cracks propagated, they connected together and broadened; near interfaces the newly formed branches were relatively narrow, which repeatedly shrank and expanded. When the cell was short-circuited, numerous cracks formed and interconnected with each other (Figure 4e), which were broadly in two orientations: radial and circumferential, as shown in Figure S1i.

**Figure 4:**
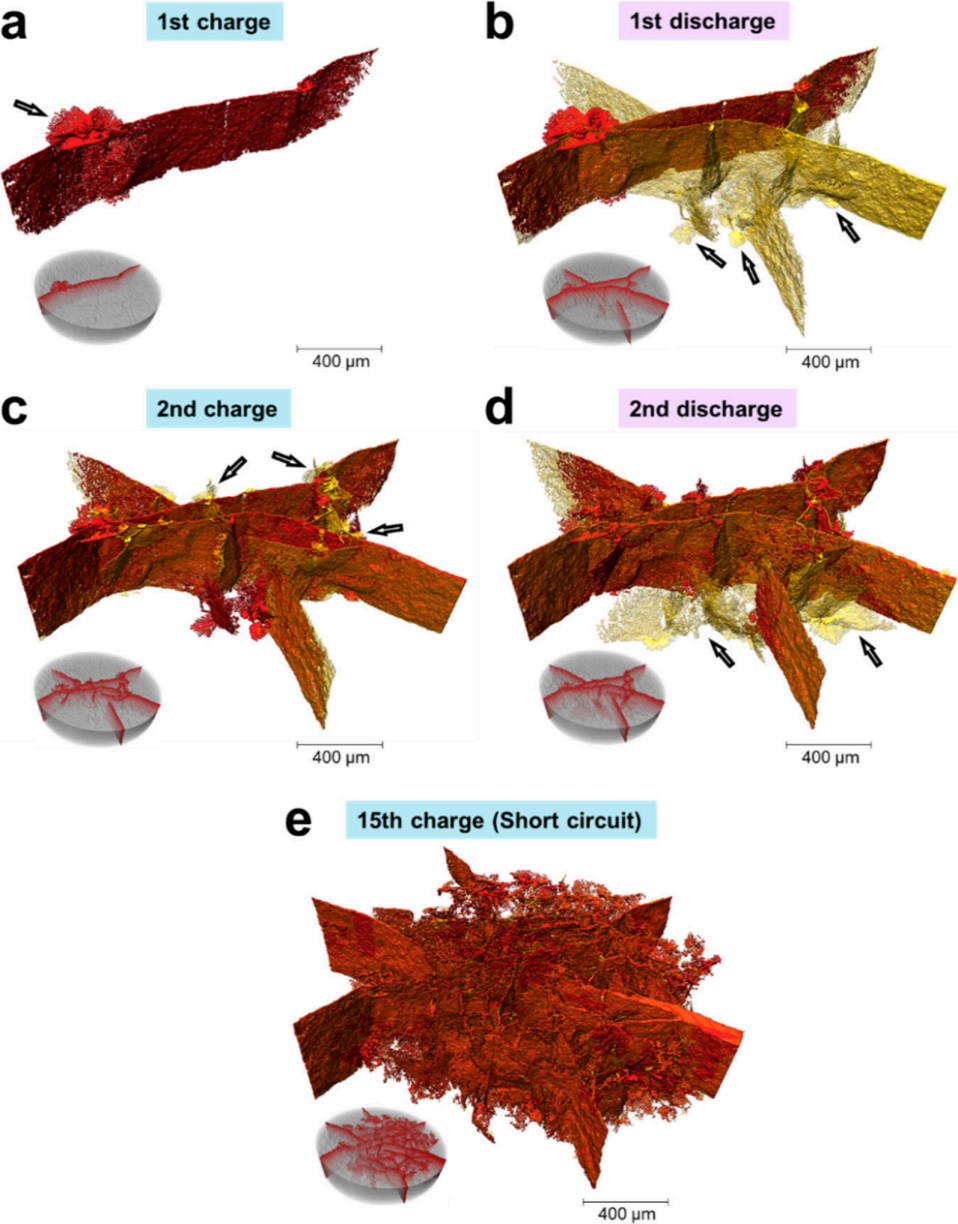
3D rendering of cracks after the 1st charging (a), 1st discharging (b), 2nd charging (c), 2nd discharging (d), and 15th charging (e), with the cracks formed in previous steps in red and the newly formed part in transparent yellow. The insets in the bottom-left corner are the 3D rendering of LPS pellet in transparent gray.

Next, the morphological changes are quantified and statistically analyzed from the tomographic image series. Figure 5a shows the crack width distribution extracted from each 3D tomogram and compared among different cycling steps. During cycling, the quantity of cracks dramatically increased, and the main peak located at ca. 3.0 μm (inset of Figure 5a) which only slightly enlarged to ca. 4.4 μm until short circuit. This illustrates, in general, that on subsequent cycling, the width of most cracks did not increase dramatically, except for isolated examples, such as the branch on top of the front views in Figure 2. This is in contrast to our previous study,^29^ where during repeated charging, cracks would visibly broaden from ca. 5 to 40 μm, and the number of cracks were much fewer. This suggested that fewer cracks formed but expanded a lot when they were driven continuously from one direction. Comparatively, in the current work, the repeatedly reversed directions of lithium deposition resulted in changing the direction of the driving force and distributions in each charging/discharging step, which induced more cracks but led to generally smaller expansion of those cracks present. Therefore, the cycling conditions of the cell can obviously influence the crack growth and crack density due to different propagation processes, which should be also considered in designing/improving solid-state batteries.

**Figure 5:**
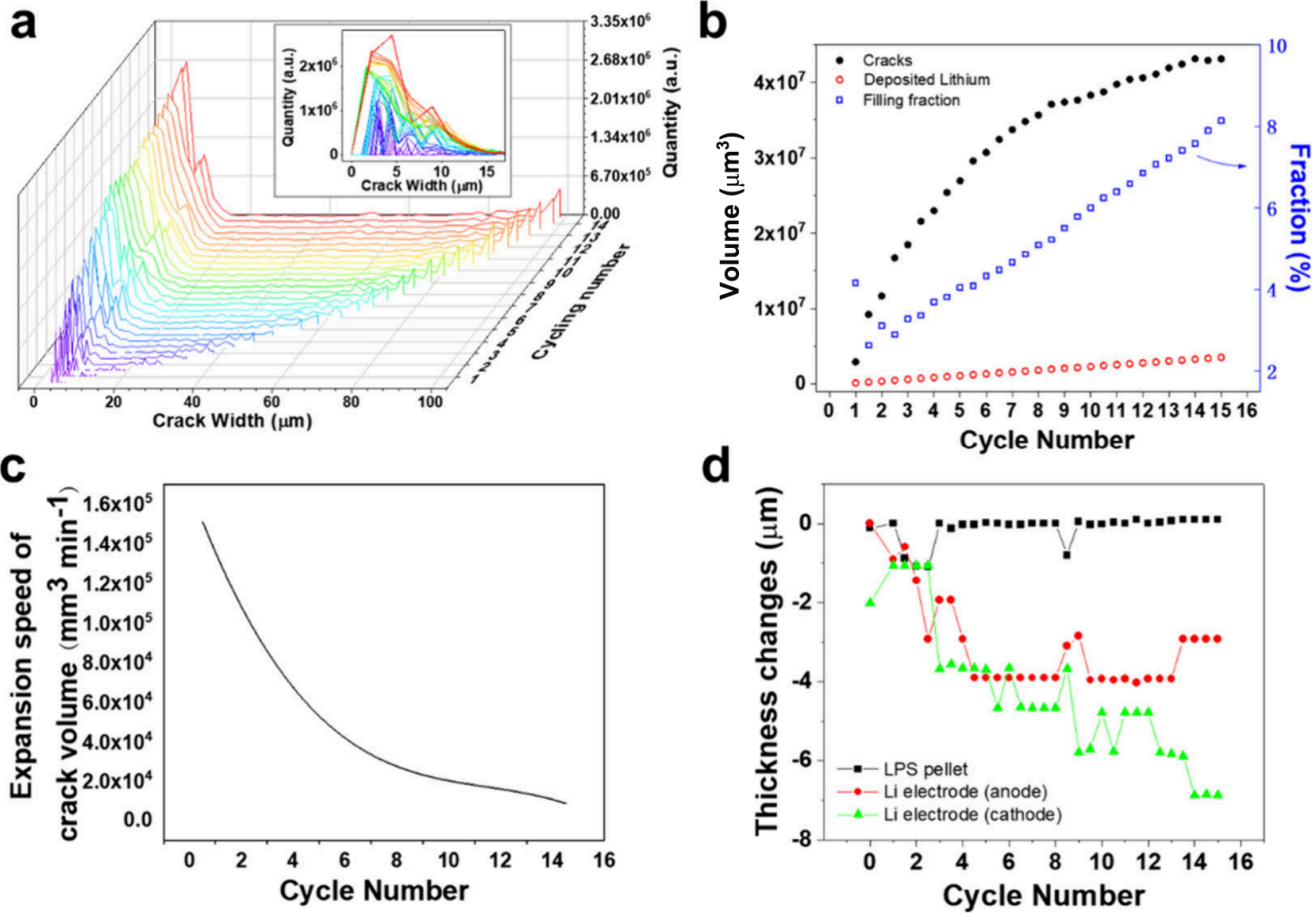
(a) Distribution of crack width after different cycling times. (b) Volume of cracks and the deposited lithium after different cycling times, and the corresponding filling fraction. (c) Plot of the speed of crack volume expansion during the whole cycling times. (d) Plot of the thicknesses change of the LPS pellet and Li electrodes during cycling. In labeling cycle number, the charging steps are set as integers and the discharging steps are set as the nearest half number (e.g., the 1st discharging is numbered as “1.5” in the plots.).

In Figure 5b, the crack volume was quantified in all tomograms and plotted versus cycle number. It increased in an approximately logarithmic fashion during the whole cycling process. Based on the charge transferred in each charging/discharging step, the volume of the deposited lithium was calculated, assuming all transferred charge cost in lithium deposition. It was proportional to the cycling time, as shown by the hollow red dots. By comparison, the volume of cracks was always much larger than that of lithium during the whole cycling process. This indicates that cracks had lots of empty space/voids inside, if taking all cracks into consideration. It should be noted that averaged values are presented in Figure 5b, so some cracks can have higher filling ratios and others lower ones. More than likely, there could be dominant pathways along specific cracks, and others were largely empty with correspondingly lower filling ratios. Additionally, if some lithium deposited at the interfaces between LPS and lithium electrodes, the amount of lithium in cracks would be less still and leave more voids within the cracks. The partial filling was also confirmed by the evolution of greyscale values in line profiles in our previous study.^29^ The filling fraction (the volume ratio of lithium to cracks) is calculated and shown by blue hollow dots in Figure 5b. It started from a relatively high value at 4.15% after the first charging, quickly dropped to 2.62% after the first discharging, and then gradually increased in subsequent cycling steps until short circuit to 8.13%. In the situation of a repetitive charging,^29^ a more significant drop in the filling fraction was observed after the cracks penetrated through the solid electrolyte; and the fraction was initially very high at 94.95% before cracks grew through the electrolyte. Therefore, during the first charging step in this case, we might expect a filling ratio much higher than 4.15% when the first crack did not penetrate the pellet yet. At the early stages of dendrite initiation, the high filling ratio indicated the cracks worked as reservoirs for lithium deposition, until they were filled up. The observed drop in filling fraction suggests an abrupt growth in cracks. In other words, the speed of crack propagation dramatically exceeded the lithium deposition. Considering the high brittleness of LPS with low fracture toughness, cracks resulted from a kind of brittle fracture which progressed very quickly. Comparatively, the lithium deposition required lithium ions to transfer under electrochemical forces, which was much slower. Therefore, the deposited lithium cannot fully fill cracks or grow simultaneously with cracks; indeed, it propagated much more slowly. As a result, the global filling ratios of lithium dendrites in cracks were greatly decreased.

More importantly, the partially filled cracks resulted in a lag of short circuit: although cracks had already penetrated the solid electrolyte after the first charging, the cell worked well with stable voltage curves. Only when the deposited lithium gradually accumulated in cracks, slowly building an electrical connection between the cathode and anode (after cycling for 15 times in this case), was the cell short-circuited. This phenomenon has also been observed in repetitive charging;^29^ however, the cell worked for a much shorter time (<7 h, compared with ca. 14 h in Figure 1b) after the solid electrolyte was penetrated. In the earlier study, the filling fraction was much higher (stable at ca. 20%) and steadily decreased while the crack volume increased in an approximately linear fashion and the crack width expanded. There are significant differences from the scenarios in this study in Figure 5b. The differences are attributed to the different cracks and lithium behavior under different electrochemical process: in the cycling test here, the reversed directions of lithium deposition stopped lithium and cracks continuously growing and triggered more cracks to form at two interfaces. Although cracks will not directly cause a short circuit, they provide preferential pathways for lithium deposition, which naturally influences the working lifetime of the cells. In general, the working time of a cell before a short circuit is influenced by both the mechanical properties of the solid electrolyte and the electrochemical test conditions.

By nonlinear fitting of the data of crack volume versus cycling time in Figure 5b (the detail of the fitting is attached in Figure S3), the expansion speed of the crack volume can be obtained from the slope of the curve, as shown in Figure 5c. As most of the cracks did not significantly expand in width, the expansion of crack volume was largely contributed from new cracks spreading in planes that were normal to the pellet surface. Therefore, the expansion speed roughly reflects the speed of crack propagation in 2D planes. It suggests that the cracks initially propagated rapidly but quickly slowed down following an exponential relationship. This behavior can be understood from a mechanical perspective: the high initial speed came from the internal stresses which accumulated inside the LPS pellet during the lithium aggregating into tiny and invisible voids and flaws at the pellet surfaces.^38,39^ However, once the cracks grew through the pellet, the stresses would be greatly released. Then, the deposited lithium, driven by the electrochemical force in cycling, added a relatively smaller force to cracks. This will be discussed further in the following section.

With the help of high-resolution X-ray CT images, the thicknesses of lithium electrodes and the LPS pellet were measured to see their changes during cycling, as shown in Figure 5d. To be accurate, the thickness was extracted from the line profiles of the image greyscale, as shown in Figure S4. The two lithium electrodes were found to be gradually compressed by ca. 4–7 μm, especially during the first five cycles, while the LPS pellet was nearly unchanged. The compression in the lithium electrodes was forced by the internal stresses from lithium deposition as well as stack pressures in the battery case from both top and bottom sides. It is also reflected in the extension of lithium layers in cross-sectional images, as indicated by a dashed line in Figure 3. In turn, the decrease in thickness and extension in lithium layers confirmed the existence of stack pressure which was maintained during the whole cycling but would be gradually released after the electrode compressed. This also ensured intimate contact at the LPS/Li interfaces and to enhance the lithium to migrate and refill voids at interfaces.

The speed of crack propagations and electrode compression can change under different packing pressures while their morphological changes and corresponding mechanisms may remain similar. While we expect the cell configuration (coin cell, cylinder cell, pouch cell, or prismatic cell) will have an influence on the behaviors of cracks inside, more investigations are needed on this, but this is beyond the scope of this work.

Historically, the lithium was assumed to fully fill in cracks in the theoretical and modeling studies;^38,40,41^ correspondingly, the processes of crack propagation along with lithium deposition were analyzed based on this premise. In our previous work^13,29^ and this work, the phenomenon of a partial filling of lithium in cracks has been observed in different samples under different cycling conditions. This indicates new electro-chemo-mechano processes in which the lithium with only a partial filling can drive and force cracks to grow in solid electrolytes.^42,43^ More recently, Claudio et al.^44^ built a theoretical framework that coupled electrochemical reactions with mechanical deformations and also reproduced the partially filled cracks with dendrites.

To get a deeper understanding, the mechanical force and electric potential field during cycling were modeled at different states of cycling, as shown in Figure 6. By utilizing the in situ X-ray CT images to build models, the scenarios in real morphologies can be investigated, not limited to simplified geometric shapes. Besides, the local contact-loss at the Li/LPS interfaces was also taken into consideration based on the observations. At the original state in Figure 6a, the modeled von-Mises stress inside the bulk of the LPS pellet was largely uniform and almost negligible. There were small stress concentrations at numerous microscopic locations along LPS/Li interfaces where the LPS surface was not perfectly smooth. These stresses were caused by the compression force in the stack pressure from top and bottom directions. These points should be considered as weak points where cracks may be preferentially generated. At this stage of cycling, the electric potential field was distributed homogeneously (Figure 6b).

**Figure 6:**
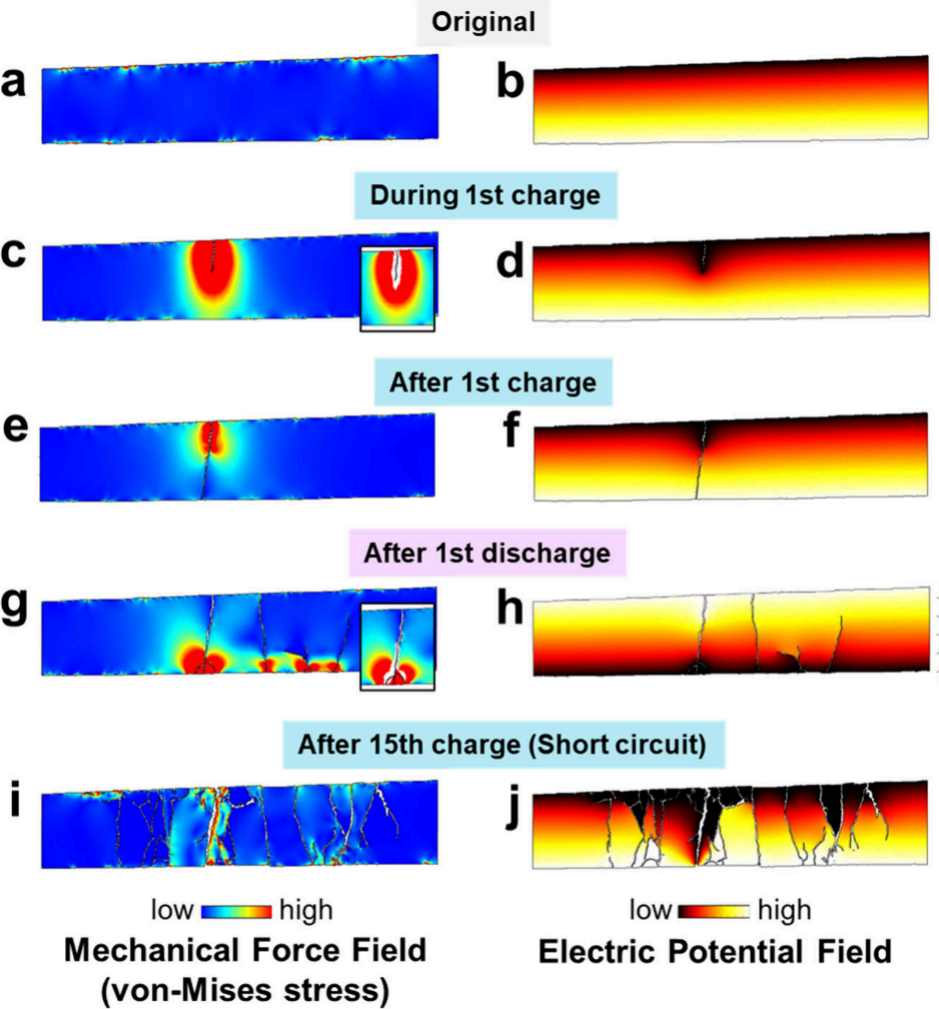
Mechanical force (von-Mises stress) field (a, c, e, g, i) and electric potential field (b, d, f, h, j) distributions in the LPS pellet at different states: (a, b) original state; (c, d) some time before 1st charging; (e, f) after 1st charging; (g, h) after 1st discharging; (i, j) after short circuit.

As the charging progressed, there was a time when cracks nucleated but did not yet grow through the LPS pellet (before the end of the first charging). The cracks should be nearly fully filled by lithium at this stage according to our previous study.^29^ In this case, this stage happened too fast to be captured, so it was inferred here by using the initial part of cracks that formed in the first charging, as shown in Figure 6c. We hypothesize that the lithium full-filling in cracks added stress on the crack walls directly, forcing them to grow in length and to open in width. The trend of deformation is shown in the inset of Figure 6c. This is in agreement with previous theoretical analyses.^38,40,41^ Meanwhile, the equipotential line in the electric potential field was curved (Figure 6d), as the electrically conducting lithium lowered the local potential, so there was a higher field strength of the electric potential at crack tips, which can promote more lithium to diffuse and deposit there. When the stress in the deposited lithium was accumulated sufficiently to fracture the LPS pellet, cracks could propagate quickly ahead of the lithium and penetrated the pellet, until the end of the first charging in this case (Figure 6 e-f); then the strain energy was largely released. As the speed of lithium deposition was much slower than the crack propagation, the new crack regions were largely unfilled. As a consequence, the cracks at this stage had a low filling ratio, and following the first cycle, a decrease in filling ratio was observed, as discussed in Figure 5b and the previous study.^29^

When the current direction was reversed in the first discharging, the lithium would preferentially deposit in the empty space at the other end of cracks and add pressure to open cracks from the other direction (i.e., the bottom side in Figure 6g). The stress was primarily located in small areas where lithium deposited near the LPS/cathode interface. Considering the whole LPS bulk as a rigid body, the pellet was forced to separate from one side, making cracks become narrower near the top side. This trend is reflected in the inset of Figure 6g. Therefore, the cracks near pellet surfaces were observed to broaden and shrink repeatedly along with cycling, as previously discussed (Videos 1 and 2 and Figure 2). In the electrical field, the equipotential lines were influenced by the deposited lithium at the bottom side, so that the field strength of electric potential was increased further in the empty part in cracks (Figure 6h). Although the increasing voids at the Li/LPS interfaces (Figure 3) did not have a significant effect on the field distribution of electric potential, they resulted in a higher current density, also enhancing the tendency of lithium deposition and filling in cracks.

In the next charging and discharging steps, a large number of cracks formed in the LPS pellet along with repeated broadening and shrinkage. The pellet as a whole, “bent” back and forth, which was forced by the lithium depositing into the partially filled cracks from two directions alternately. During these processes, the mechanical stress was much released, especially after the pellet was fully penetrated through the thickness. However, the electric field was increasingly concentrated in the empty (unfilled) part in the cracks because of the decreased interfacial area and the decreased distance from the unfilled position in cracks to the counter electrode. Finally, when the cell was short-circuited, there was a small stress, broadening the crack width (Figure 6i), and a very distorted field distribution of electric potential (Figure 6j). The resultant displacement of the LPS fragments in each cycling step was calculated by digital volume correlation (DVC) analysis, as clearly shown in Figure S5. Generally, the partial filling of lithium inside cracks happened under the combined effects of the electrochemical deposition of lithium and the mechanical growth of cracks. During cycling, the morphological changes of cracks and lithium redistributed both the force fields and the electric potential fields in each step; in turn, these field distributions promoted crack growth and lithium deposition further.

## Conclusion

In this work, the degradation processes of LPS solid electrolyte in a whole cycling process until short circuit were tracked by imaging its dynamic morphological evolution in 3D, utilizing in situ X-ray CT. With the help of high-flux synchrotron radiation as well as careful material selection and bespoke cell design, the spatial resolution was improved to 0.8 μm voxel size; meanwhile, a sufficiently high temporal resolution was realized to capture dynamic changes without any disturbance or movement to the sample. With the current direction changed repeatedly, thin sheet-like cracks alternately initiated from two LPS/Li interfaces; correspondingly, the LPS pellet was forced to “bend” back and forth. The crack propagation slowed down in an exponential trend with time. Through image processing and statistically quantitative analysis, the filling ratios of lithium deposition in cracks were found to drop dramatically and then increase slightly. Correspondingly, the two layers of lithium electrodes were mainly compressed during the first five cycles by ca. 4–7 μm. Compared with that in a repetitive charging process, the behaviors of crack growth and lithium filling ratio were different. Finally, the distributions of both mechanical force field and electric potential field were modeled using real morphologies, and their evolution was mapped at different states of cycling. The strain energy was largely released just when the solid electrolyte was first penetrated, while lithium deposition and crack growth continued under the coupled effect of these two fields until a final short.

These results show a fast degradation of the solid electrolyte happened in initial cycling stages, including fast crack propagation, high lithium filling ratio, and large electrode compression. This highlights the significance of the initial states and inspires more thoughts for prevention or mitigation strategies, especially from inhibiting cracks and modifications of the mechanical properties of the solid electrolyte.

## Experimental Section

### Cell Fabrication

Li_3_PS_4_ powder (MSE Supplies LLC, USA) was cold pressed in an Ar-filled glovebox, into a pellet with a thickness ca. 0.6 mm and a diameter of ca. 2 mm. The symmetrical Li/LPS/Li cell was assembled in modified PFA Swagelok straight unions (PFA-220–6, Swagelok, UK), with ca. 0.8 mm thick lithium foil pressing onto both sides of the LPS pellet.

### Synchrotron X-ray Tomography

In situ experiments were conducted on the I13-2 Diamond-Manchester Branchline at Diamond Light Source (Harwell, UK). The X-ray beam used for imaging was a filtered pink beam with an energy range of 18–24 keV. The spectral mean energy was 22.3 keV. The spectrum was generated by using an ID gap of 5.0 mm and the following filters with respective thicknesses *t*: pyrolytic graphite, *t* = 1.34 mm; aluminum, *t* = 2.1 mm; silver, *t* = 35 μm; palladium, *t* = 42 μm. The detector was a PCO.Edge 5.5 detector with 4× objective lens for 8× total optical magnification, resulting in a pixel size of ca. 0.8 μm and a field of view of ca. 2.1× 1.8 mm. During each tomographic acquisition, a total of 2400 projections were collected over 180° angular range with an exposure time of 0.2 s per frame. Before the in situ test, the samples were imaged under the same settings to ensure its intactness without any cracking. Electrochemical processes were stopped during imaging to ensure a consistent state of charge through the scan. The projections were corrected for lens distortion,^45^ and a ring-removal algorithm^46^ was applied. They were reconstructed using the SAVU framework^47^ and the tomopy gridrec algorithm.^48^

The tomograms were processed in Avizo (Thermo Fisher Scientific, Waltham, Massachusetts, USA) through filtering and segmentation. The region of the LPS pellet was isolated from the whole volume. The width of cracks was quantified and counted in each tomogram to get its distribution. The volume of cracks was extracted and compared at different plating times.

### Electrochemistry

Between each tomographic acquisition, the cell was charged with a potentiostat (Interface 1000E, Gamry Instruments) at a constant current density of 0.15 mA cm^–2^ at room temperature. It was kept on the beamline sample stage without any movement. The beam shutter was closed during each charge/plating step.

### DVC Analysis

DVC analysis was carried out in DaVis (V10.1, LaVision, Germany) on the in situ tomograms to retrieve the displacement and strain in the LPS pellet. The tomograms were cropped to remove empty volume outside of LPS pellet. A multipass scheme was used to correlate volume by subvolumes of 32^3^, 16^3^, 12^3^ and 8^3^ voxels with a 75% overlap. Each tomogram was sorted by cycling numbers and correlated to its predecessor. Thus, the resulting displacement and strain field describes changes between tomograms after adjacent cycling times. Vectors were colored based on the displacement range in each image.

### Simulation Methods

The mechanical force field and electric potential field in the LPS solid electrolyte were investigated by COMSOL Multiphysics 5.5 based on finite element method analysis. The in situ X-ray CT results were imported in a format of 2D images to build the 2D models, so that the morphologies in modeling were the same as that obtained in the experiments. In the mechanics analysis, the mechanical parameters were obtained from the reported values in the literature which are listed in the Supporting Information. The pressure was applied vertically from the top and bottom of the LPS pellet. The plastic deformation was assumed to be negligibly small. In electrostatics analysis, with the growth and filling of lithium dendrites in cracks, the effective conductivity at those areas turned to the lithium conductivity. The cathode potential was set as 1.0 V, and the anode side was 0 V.

## Supplementary Material







## Notes

Additional X-ray CT images (Figures S1 and S2); details of data processing and calculations (Figures S3 and S4); DVC analysis (Figure S5); simulation details (Table S1) (PDF)
Video 1: top view of morphological evolution of solid electrolyte during the cycling process (AVI)
Video 2: side view of morphological evolution of solid electrolyte during the cycling process (AVI)

## ◧

Institute of New Energy Materials Chemistry, School of Materials Science and Engineering, Nankai University, Tianjin, 300350, China

## ○

Helmholtz-Zentrum Hereon, Institute of Materials Physics, 21502 Geesthacht, Germany
