# Supplementary material for: Fast Degradation of Solid Electrolyte in Initial Cycling Processes, Tracked in 3D by Synchrotron X‑ray Computed Tomography
Source: ACS Nano. 2025 May 28;19(22):20516–25. doi: 10.1021/acsnano.4c17739 (PMC12164515; doi:10.1021/acsnano.4c17739)
Supplement: Supplementary file 1 [file nn4c17739_si_001.pdf]

Supplemental Information

Fast Degradation of Solid Electrolyte in Initial Cycling Processes, tracked in 3D by Synchrotron X-ray Computed Tomography

Shuai Hao[1,2,3], Sohrab R. Daemi[2,3], Thomas M. M. Heenan[2,3], Wenjia Du[2,3,6], Malte Storm[4,5], Mohamed Al-Hada [2,3,4], Christoph Rau[4], Dan J. L. Brett[2,3], Paul R. Shearing[2,3,6]*

[1]*present address: Institute of New Energy Materials Chemistry, School of Materials Science and Engineering, Nankai University, Tianjin, 300350, China.*

[2]*Electrochemical Innovation Lab, Department of Chemical Engineering, University College London, London WC1E 7JE, UK*

[3]*The Faraday Institution, Quad One, Harwell Science and Innovation Campus, Didcot OX11 0RA, UK*

[4]*Diamond Light Source Ltd, Harwell Science & Innovation Campus, Didcot, Oxfordshire OX11 0DE, United Kingdom*

[5]*present address: Helmholtz-Zentrum Hereon, Institute of Materials Physics, 21502 Geesthacht, Germany*

[6]*The ZERO Institute, University of Oxford, Holywell House, Osney Mead, Oxford, OX2 0ES*

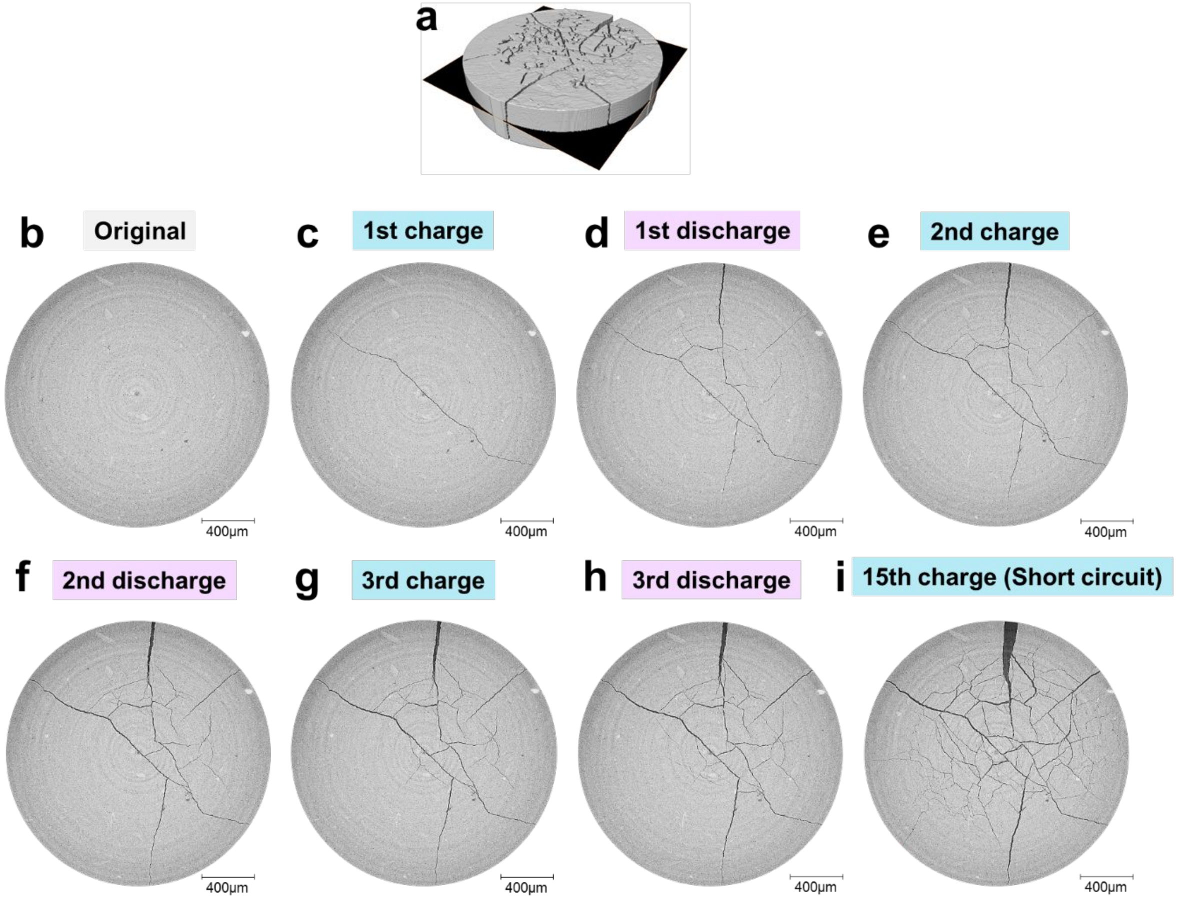

Fig S1. 2D orthogonal slices extracted from 3D tomogram as illustrated in (a, the 3D rendered volume of LPS pellet after 15th charging), comparing them at original state (b) and after 1st (charging:

c; discharging: d), 2nd (charging: e; discharging: f), 3rd (charging: g; discharging: h) and 15th (charging: i) cycling.

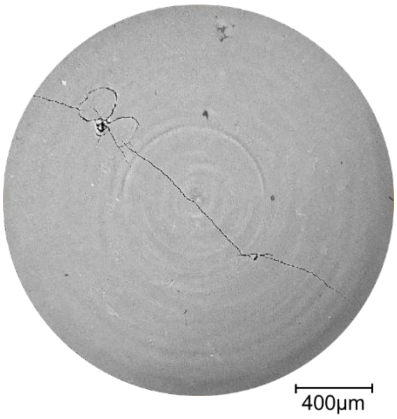

Fig S2. 2D slices near the interface with anode

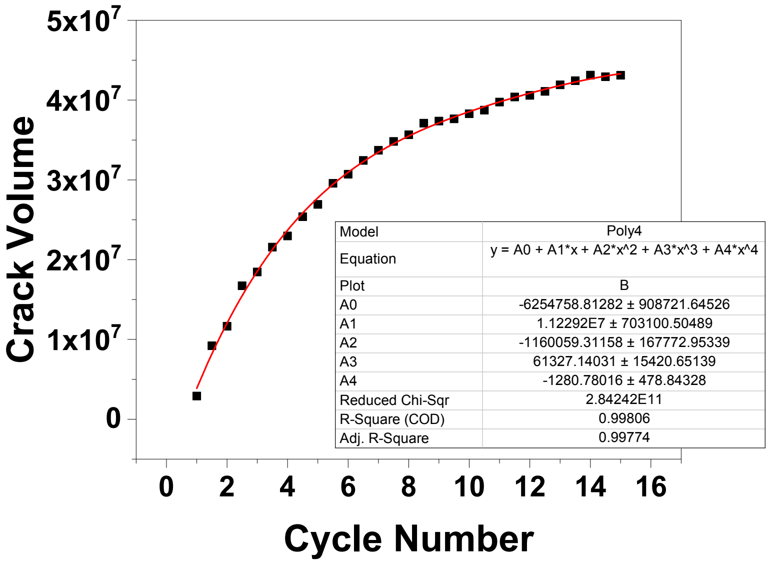

Fig S3. Non-linear fitting the changes of crack volume versus cycling time

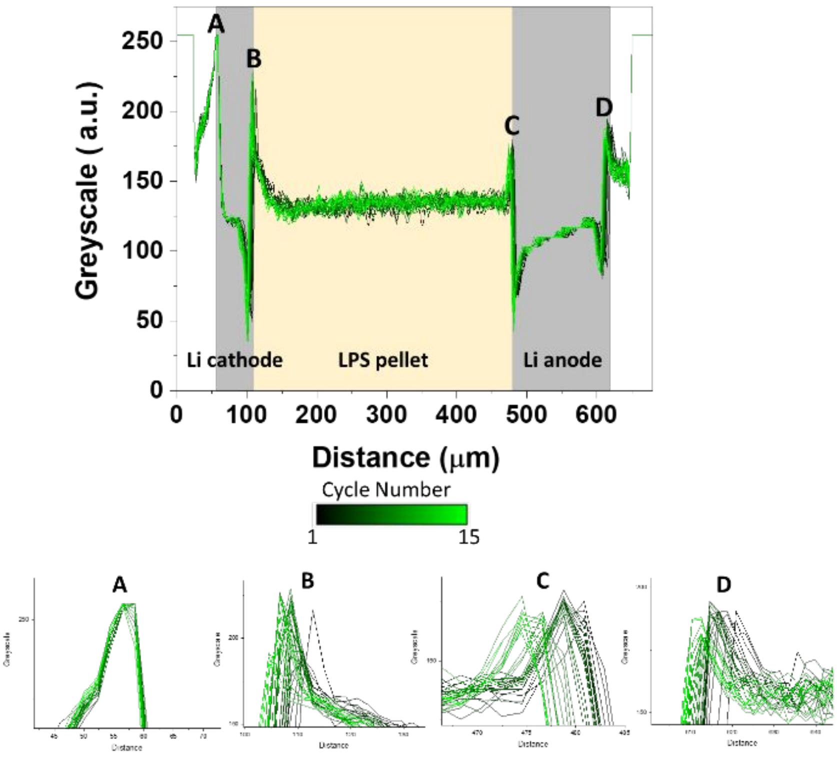

Fig S4. The line profiles of the image greyscale, extracted from the same path perpendicular to the Li/LPS/Li layers

Thickness changes were extracted from the greyscale of a line in "Side view". There are obvious different X-ray attenuations between Li electrodes and the LPS pellet. In order to measure precisely, we selected the greyscale peaks as segmentation points (labelled as A, B, C and D in the figure and partial magnifications). Then, the thickness of electrolyte and electrodes were calculated from the distance of two peaks. And their changes in different cycles were analysed statistically.

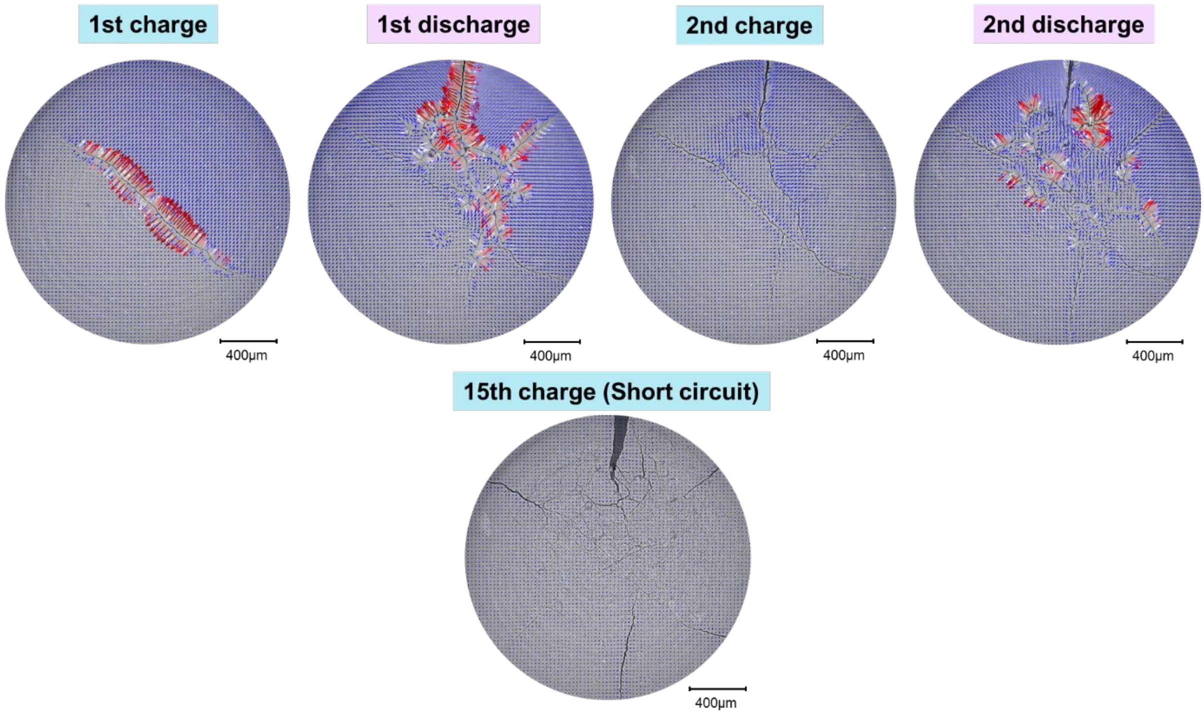

Fig S5. The displacement along cracks calculated in DVC analysis, viewed at the same position of sample after the 1st charging, 1st discharging, 2nd charging, 2nd discharging and short circuit.

Simulation details

Table S1. Summary of material properties used in the modelling

Parameter	Value	References
Young's modulus	28.9 GPa	1
Poisson's ratio	0.27	1
Density	1.87 g/cm^3	2

The in-situ X-ray CT results were imported in a format of 2D images to build the 2D models. In mechanics analysis, the mechanical parameters were obtained from the reported values, as listed in Table S1. The pressure was applied vertically from the top and bottom of LPS pellets, meanwhile it was also added vertically to some crack edges where they were filled with dendrites. The top/bottom edges of LPS pellet and crack edges were selected as boundaries for applied pressures. All stresses and strain were set to be in plane. The simulation domain was processes by triangle meshes, and "Adaptive Mesh Refinement" was utilized to small regions with curved edges. Therefore, the inhomogeneous distributions of stresses on the uneven surface were reflected, as magnification in Figure S6.

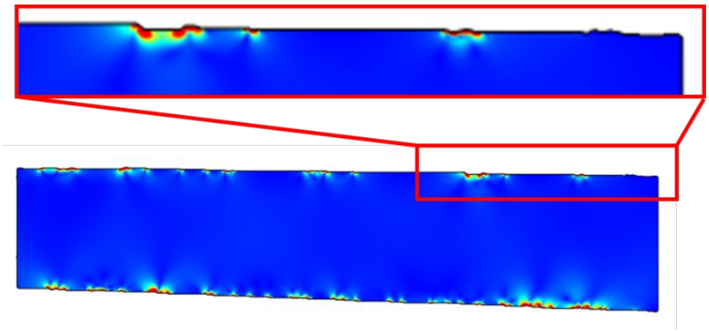

Fig S6. Partial magnification of the mechanical force field distributions

1. Yang, Y.; Wu, Q.; Cui, Y.; Chen, Y.; Shi, S.; Wang, R. Z.; Yan, H., Elastic Properties, Defect Thermodynamics, Electrochemical Window, Phase Stability, and Li(+) Mobility of Li3PS4: Insights from First-Principles Calculations. *ACS Appl Mater Interfaces* **2016,** *8* (38), 25229-42.
2. Baranowski, L. L.; Heveran, C. M.; Ferguson, V. L.; Stoldt, C. R., Multi-Scale Mechanical Behavior of the Li3PS4 Solid-Phase Electrolyte. *ACS Appl Mater Interfaces* **2016,** *8* (43), 29573-29579.
